# Strengthening the Surveillance of Antimicrobial Resistance in India Using Integrative Technologies

**DOI:** 10.3389/fpubh.2022.861888

**Published:** 2022-04-04

**Authors:** Jasleen Kaur, Jasmine Kaur, Ajay Singh Dhama, Shelja Jindal, Kamini Walia, Harpreet Singh

**Affiliations:** ^1^Division of Biomedical Informatics, Indian Council of Medical Research, New Delhi, India; ^2^Epidemiology and Communicable Diseases Division, Indian Council of Medical Research, New Delhi, India

**Keywords:** women, public health, infectious disease, surveillance, interoperability, antimicrobial resistance

## Abstract

**Background:**

The antimicrobial resistance (AMR) situation in India is alarming. In the absence of newer antibiotics, the best possible approach is to efficiently use the existing antimicrobials through surveillance of resistance. The data generated by AMR surveillance across the country has immense potential to drive policy decisions. However, this data is available in a variety of sources. It is imperative to have tools to integrate the data generated across the country into a single data repository.

**Methods:**

An ensemble of tools (*i*-AMRSS, *i*-DIA, and *i*-AMRIT) have been designed and developed by the data management team at the Indian Council of Medical Research (ICMR) to strengthen surveillance of antimicrobial resistance in India.

**Results:**

The *i*-AMRSS is a web-based ICMR's AMR surveillance system, collecting data from tertiary care centers across the country and sending it to the one-stop data repository. The *i*-DIA is a web-based API that simplifies the AMR data interoperability by seamlessly importing most of the LIS / HIS data from CSV files into a central, one-stop data repository. The *i*-AMRIT is a standalone ICMR's AMR surveillance system using integrative technologies, collecting data from all the labs across the country and sending the lab-specific cumulative data to the one-stop data repository.

**Discussion:**

The tools are being used in ICMR's AMR Network and have collected over 0.4 million patient records to date. The complete system is presently being used to capture human susceptibility testing data and can be extended for capturing data using the ‘One Health' approach. The authors plan to make the system compliant with FHIR standards to enable interoperability with other countries.

## Introduction

Antimicrobial resistance (AMR) is one of the top 10 public health threats the world is facing today (1). AMR does not discriminate, but the burden majorly falls on low-and-middle income group countries. The AMR situation in India is alarming. India is among the countries having the highest antibiotic consumption and resistance rates (2, 3). The ongoing pandemic has led to the continuing high rate of antibiotic prescriptions, which may lead to even worse AMR situations post pandemic (4, 5).

In July 2018, the Ministry of Health and Family Welfare and the National Center for Disease Control (NCDC) released Guidance for developing State Action Plans for Antimicrobial Resistance (SAP CAR) (6). As per the document, the basic framework for developing the State action plan is derived from six strategic priorities of NAP-AMR (7). One of the essential objectives of NAP includes establishing a laboratory-based AMR surveillance system in the country to generate quality surveillance data (6).

Currently, India has three large AMR networks operating to collect AMR data from tertiary hospital settings. The National Center for Disease Control (NCDC) network is now the focal point for implementing the National Programme on Containment of Anti-Microbial Resistance (AMR) (8) and collects data from a network of ~60 tertiary hospitals. The NCDC network uses WHONET, a standalone open-source windows-based software for data collection. The Hospital Acquired Infection (HAI) Surveillance Network (9) is a collaborative effort by All India Institute of Medical Sciences (AIIMS), New Delhi, Centers for Disease Control and Prevention (CDC) (10) and the Indian Council of Medical Research (ICMR) (11) to strengthen national capacity for surveillance of HAIs. The network has developed a web-based tool for data collection. The Indian Council of Medical Research (ICMR) has established a network (12) collecting data on AMR since 2013. The network uses an in-house web-based solution for collecting, managing, and analyzing data from the network, which is spread across 30 public tertiary care hospitals and some private hospitals and labs (e.g., SRL, Lal Path labs, etc.) across India.

With the worsening situation and growing awareness among the states, huge volumes of AMR data are expected to be generated with the following characteristics: (i) volume—AMR data (13) must be reported by about 200,000 public health facilities across the country. This amount will increase with the inclusion of data from gene sequencing; (ii) variety—Data will come in a variety of formats from both paper and computer-based systems, including databases, emails, photographs, spreadsheets, PDFs, hospital and laboratory information systems, and other diagnostic devices; (iii) velocity—Using the present ICMR (Indian Council of Medical Research) AMR surveillance system as an example, data from ~25+ hospitals is growing at a rate of 7,000–10,000 antibiograms each month. The pace of data will be phenomenal as data begins to flow from all locations across the country; and, (iv) variability—Considering surveillance data may come from a variety of sources, including humans, animals, and agriculture, and because resistance is a very dynamic phenomenon, there will be a lot of variation in the data. As a result, India will produce “big data.” In order to use this data to drive decision making, it is essential to have tools that can capture/integrate data from varied sources into a one-stop repository.

**Figure 1:**
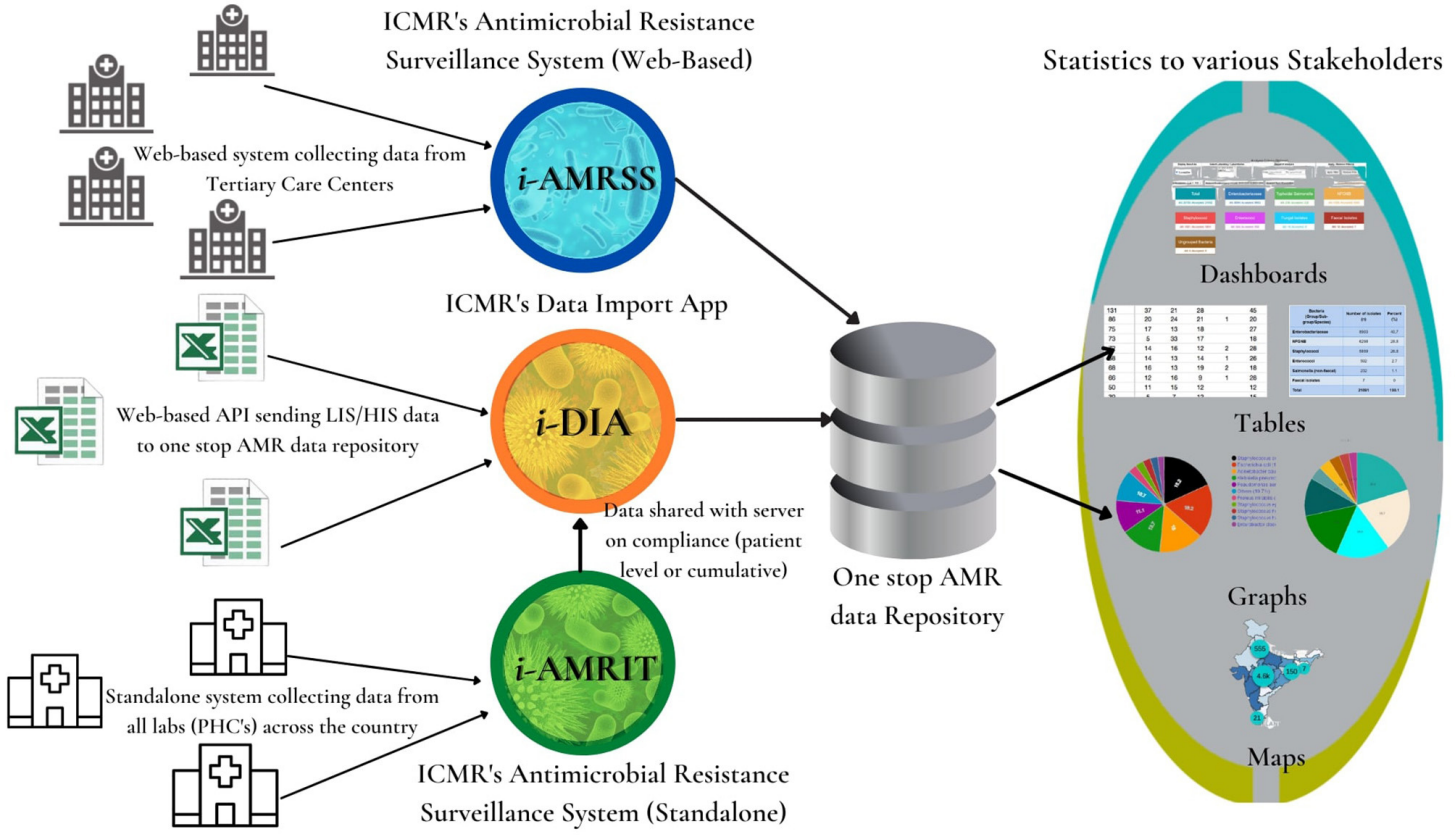
Ensemble of tools for strengthening the surveillance of antimicrobial resistance in India using integrative technologies.

This paper highlights the design and development of an ensemble of tools (as shown in Figure 1) for strengthening the surveillance of AMR in India using integrative technologies. As shown in Figure 1, the *i*-AMRSS (14–16), *i*-DIA (17, 18), and *i*-AMRIT (19) are three different tools of an advanced AMR surveillance system connected in the following way: (a) the *i*-AMRSS (14–16) is a web-based ICMR's antimicrobial resistance surveillance system collecting data (through the built-in integrated form) from tertiary care centers across the country and sending it to the one-stop AMR data repository; (b) the *i*-DIA (17, 18) is a web-based API that simplifies AMR data interoperability by seamlessly importing the majority of LIS / HIS data from CSV files into a central one-stop AMR data repository; (c) the *i*-AMRIT (19) is a standalone ICMR's antimicrobial resistance surveillance system using integrative technologies, collecting data from all the labs (PHC's) across the country and sending the lab-specific cumulative data to the one-stop AMR data repository via *i*-DIA (17, 18) web API.

## Methods

### System Architecture and Technology Used

#### *i*-AMRSS (Web-Based Tool)

The ICMR's Antimicrobial Resistance Surveillance system (*i*-AMRSS) (14–16) is an open-source, innovative web-based tool for gathering, maintaining, and evaluating the AMR data collected from different tertiary care centers across the country. The tool has been deployed in the hospitals, which are a part of ICMR's AMR Surveillance Network and has collected ~0.4 million patient records till date. The *i*-AMRSS tool can gather data provided by a variety of test methodologies, including disc diffusion, MIC, and automated testing. The data gathering module of *i-*AMRSS tool enables the data entry operators to submit the patient's data via a built-in integrated form or upload the bulk data through a web-based API (*i*-DIA) using CVS/Excel. The web-based data gathering module is divided into four sections; (a) patient-information; (b) hospital-information; (c) sample-information; and (d) susceptibility testing results.

The data validation module allows the experts to verify all the data entered into the *i*-AMRSS system for each organism class. Previously, the data was manually validated by a group of experts. However, the current version has built-in auto-validation rules which highlight any unusual resistance patterns in the data. The data analytics module in the *i*-AMRSS system enables an in-depth study of the information recorded in the system.

**Figure 2:**
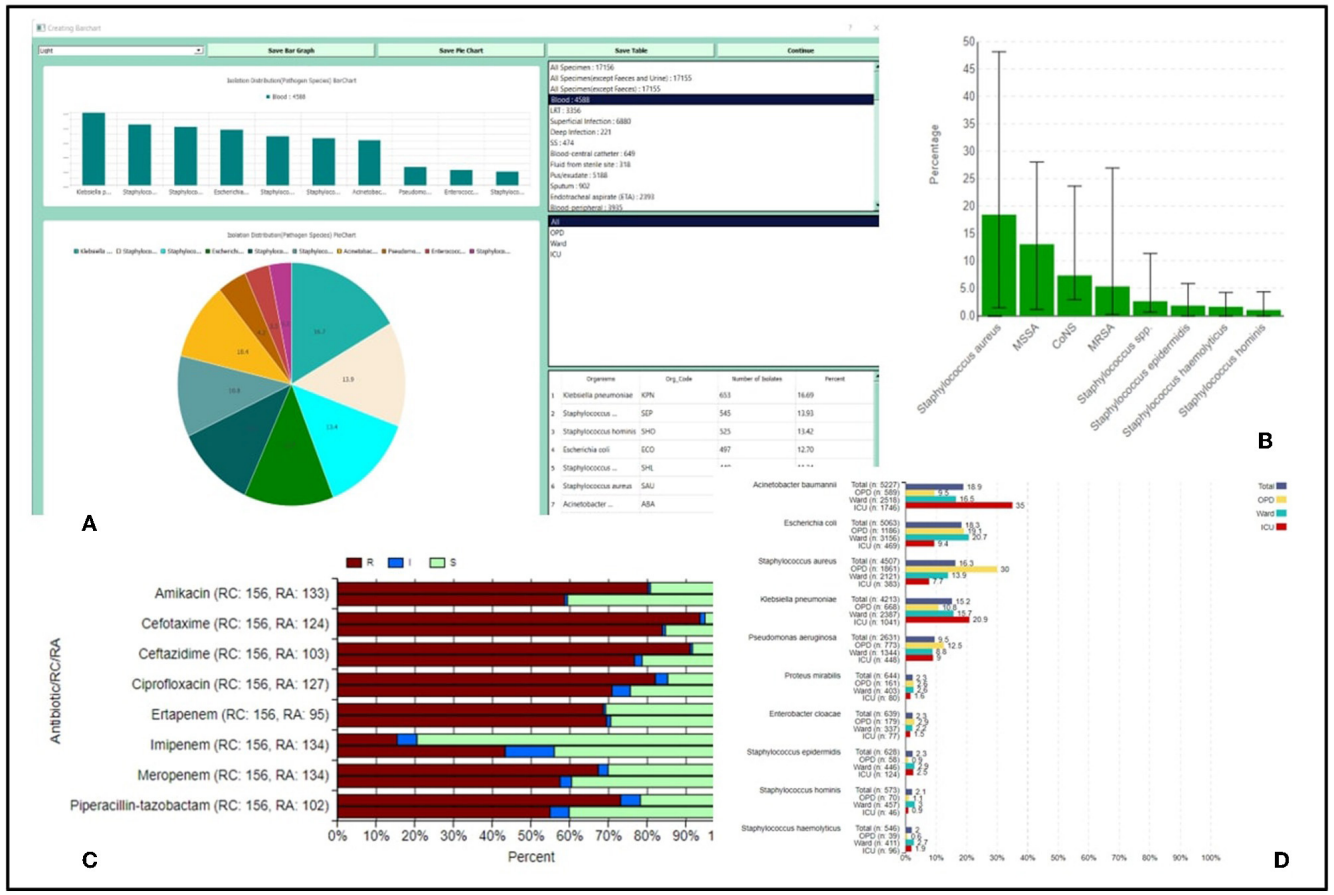
**(A)** Isolation distribution of top 10 organisms isolated from specimens and organism groups based upon the location type; **(B)** Bar graphs representing isolation percentage of Staphylococcus species across all regional centers; **(C)** Stacked bar charts giving a comparative account of RIS in a regional center as compared to rest regional centers in the network; **(D)** Bar charts location-wise resistance percentage of antibiotics tested for *Klebsellia pneumoniae* isolated from all samples.

The analysis is displayed to the user in the form of pie charts, bar charts, stacked bar charts, and tables, demonstrating the comparable and specific isolation percentages and susceptibility variations as depicted in Figure 2.

#### *i*-DIA (Web-Based API)

The ICMR's Data import app (*i*-DIA) (17, 18) is a login based, browser independent web API. The *i*-DIA is a protected and configurable framework that includes the features for building the hospital-specific customized configuration files using the mapping API and sending the large LIS/HIS data from excel/CSV files to a one-stop AMR data repository.

#### *i*-AMRIT (Standalone Tool)

The ICMR's Antimicrobial Resistance Surveillance system using integrative technologies (*i*-AMRIT) (19) is a platform-independent, API enabled standalone system, that can consolidate data from all the labs (PHC's) across the country. The *i*-AMRIT software is available for download at amrit.icmr.org.in, with AMRIT.exe included in the downloaded AMRIT.zip package.

The tools have been designed and developed using standard open-source technologies (PHP and JavaScript for *i*-AMRSS and Python for *i*-DIA and *i*-AMRIT) described in detail in the software papers. The different tech stacks were used to simulate the diversity in the data management software's available across the country.

### Data Validation

Initially, experts validated all the data uploaded from various tools to a one-stop AMR data repository. Each expert was allocated to a group of organisms for which the expert could check, track and analyze all the data. The expert also received warnings for any unexpected resistance patterns based on the pre-defined rules in the system. However, this required lot of time and efforts on the part of experts. Based on their experiences and guidelines for unacceptable susceptibility, intrinsic resistances etc., the experts formulated new set of rules for automated validated of records. The auto-validation module automatically checks for any unacceptable patterns and highlight the records that require interventions.

### Data Security and Confidentiality

The data security is ensured through the web-based *i*-AMRSS system's role-based access, which allows each stakeholder to see the dashboard panels applicable to their role. In addition, the built tool maintains an audit trail for each data that is submitted and confirmed. The web-based (*i*-AMRSS) and standalone (*i*-AMRIT) systems do not capture sensitive data of patients like addresses or phone numbers, ensuring patient's anonymity. Additionally, the *i*-AMRIT executes as a standalone system within a local hospital and is protected by the local data security, thus there are no concerns of individual patient data being accessed. The data security of the web API (*i*-DIA) is ensured by authentication server access, with the individual users having access to and seeing their own hospital's specific configuration files. In the central one-stop AMR data repository, data security is ensured by the strong data encryption for Patient ID and Sample ID.

## Results, Discussions, and Future Directions

The sources of AMR data can be broadly divided into three components based on AMR data availability (a) sites with data already in a one stop repository i.e., the sites in the ICMR AMR network using *i*-AMRSS for collecting the data (b) sites with data in their own LIS/HIS systems (c) sites with data in excel sheets/registers with no standardized data collection tool. In order to use AMR data to drive policy decisions, complete representation of the country is import. Thus, it is essential to ensure interoperability and integration among all the data sources in the country. The current paper describes tools that can be used for managing data from all the three sites.

The *i*-AMRSS can be used in places with good internet connectivity, the *i*-DIA to import data from any other software, and the *i*-AMRIT for sites that do not use any surveillance tool. The described tools will ensure a seamless flow of information from various data sources and its dissemination to various stakeholders for formulating guidelines and policies on AM consumption. The detailed system architecture and brief literature survey for each tool is given and described in the independent papers of these tools (14, 15, 17). The Real-time analysis of data will ensure the impact of policies is communicated to the policy-makers so that necessary steps can be taken in the right direction.

All of the discussed tools (*i*-AMRSS, *i*-DIA, *i*-AMRIT) are currently being piloted in ICMR's AMR Network (20) (with more than 25 tertiary-care hospitals across India). Over 0.4 million patient records have been collected so far by the one-stop AMR data repository. The complete system is currently being used to collect the human susceptibility testing data; however, it can be extended for AMR surveillance using the ‘One Health' approach. Some of the future developments may include making it compliant with FHIR standards to enable interoperability across countries as well. Also, developing and integrating built-in early warning systems to alarm each site for any budding outbreaks.

## Data Availability Statement

The data is available in the form of reports on the ICMR-AMR website (https://iamrsn.icmr.org.in/index.php/resources/amr-icmr-data). The funding agency has provided restricted access to the complete data. Any request for the complete data can be submitted to the funding agency and the data can be made available if approved by the competent authorities.

## Ethics Statement

Ethical review and approval was not required for the study of human participants in accordance with the local legislation and institutional requirements.

## Author Contributions

HS has conceptualized the work. JaslK and JasmK are the lead writers of the manuscript and conceptualized and designed the figure. AD and JasmK are involved in the development of *i*-AMRSS tool where AD is the lead developer. JaslK, SJ, and JasmK are involved in the development of *i*-DIA and *i*-AMRIT tools where JaslK is the lead developer. JasmK is involved in data retrieval and analysis, generating annual reports, submitting data to GLASS and conducting trainings/workshops for the research staff involved in using these tools. HS and KW have critically reviewed the manuscript. All authors listed have made a substantial, direct, and intellectual contribution to the work and approved it for publication.

## Funding

This project was funded by the Indian Council of Medical Research, New Delhi with grant number AMR/244/2020-ECD-II.

## Conflict of Interest

The authors declare that the research was conducted in the absence of any commercial or financial relationships that could be construed as a potential conflict of interest.

## Publisher's Note

All claims expressed in this article are solely those of the authors and do not necessarily represent those of their affiliated organizations, or those of the publisher, the editors and the reviewers. Any product that may be evaluated in this article, or claim that may be made by its manufacturer, is not guaranteed or endorsed by the publisher.

## Abbreviations

One Health: Approach that recognizes that the health of people is closely connected to the health of animals and our shared environment
HL7: Health Level Seven
FHIR: Fast Healthcare Interoperability Resources
*i*-AMRSS: ICMR's Antimicrobial Resistance Surveillance system
*i*-DIA: ICMR's Data import app
*i*-AMRIT: ICMR's Antimicrobial Resistance Surveillance system using integrative technologies
WHONET: Software for the Surveillance of Antimicrobial Susceptibility.
